# The circRNA-miRNA/RBP regulatory network in myocardial infarction

**DOI:** 10.3389/fphar.2022.941123

**Published:** 2022-07-18

**Authors:** Lei Zhang, Yuan Zhang, Fei Yu, Xin Li, Huijuan Gao, Peifeng Li

**Affiliations:** Institute for Translational Medicine, The Affiliated Hospital of Qingdao University, Qingdao University, Qingdao, China

**Keywords:** myocardial infarction, circular RNA, regulatory pathway, miRNA sponge, clinical application

## Abstract

Myocardial infarction (MI) is a serious heart disease that causes high mortality rate worldwide. Noncoding RNAs are widely involved in the pathogenesis of MI. Circular RNAs (circRNAs) are recently validated to be crucial modulators of MI. CircRNAs are circularized RNAs with covalently closed loops, which make them stable under various conditions. CircRNAs can function by different mechanisms, such as serving as sponges of microRNAs (miRNAs) and RNA-binding proteins (RBPs), regulating mRNA transcription, and encoding peptides. Among these mechanisms, sponging miRNAs/RBPs is the main pathway. In this paper, we systematically review the current knowledge on the properties and action modes of circRNAs, elaborate on the roles of the circRNA-miRNA/RBP network in MI, and explore the value of circRNAs in MI diagnosis and clinical therapies. CircRNAs are widely involved in MI. CircRNAs have many advantages, such as stability, specificity, and wide distribution, which imply that circRNAs have a great potential to act as biomarkers for MI diagnosis and prognosis.

## Introduction

Myocardial Infarction (MI), also called a heart attack, is caused by blocking of blood flow in coronary arteries. The blockage (atherosclerotic plaque) comprises fat, cholesterol, and other substances (Lu et al., 2015), where rupture can damage some heart muscles and result in myocardial ischemia or heart failure (HF) (Lu et al., 2015). Thus, MI can be lethal and significantly burden families and the general population. Many physiological and pathological processes are involved in MI, such as apoptosis, autophagy, myocardial ischemia/reperfusion (I/R), and cardiac fibrosis. MI diagnosis and treatment have dramatically improved over the past years. However, its incidence and mortality rates continue to increase because of the lack of effective therapeutic targets. Therefore, efforts should be made to discover and unveil underlying mechanisms, thus providing new avenues for MI diagnosis and treatment.

Noncoding RNAs are known to participate in MI pathogenesis. MicroRNAs (miRNAs) have been extensively studied, and the underlying mechanisms have been well documented (Li et al., 2019; Zhang et al., 2020a). Recently, other noncoding RNAs have also been shown to play crucial roles in MI (Zhang M. et al., 2019; Zhang Y. et al., 2019; Bai et al., 2020; Sun et al., 2021). Circular RNAs (circRNAs) are a class of circularized RNAs with a length of ∼500bp (Hansen et al., 2013). They were considered non-functional when first discovered in plant viruses (Kolakofsky, 1976). However, with advancements of technology and in-depth research, the biological functions of circRNAs were uncovered. Most circRNAs are located in the cytoplasm, with only a small number existing in nucleus (Hansen et al., 2013), which are consistent with their functional roles. CircRNAs can bind to miRNAs or RNA-binding proteins (RBPs) to influence the biological activity of downstream targets (Liu et al., 2022), thereby modulating a lot of signaling pathways.

Numerous studies have revealed the multifunctional roles of circRNAs in the pathogenesis of various diseases, including MI (Deng et al., 2016; Geng et al., 2016; Cai et al., 2019; Garikipati et al., 2019). CircRNAs are stable under different conditions due to their covalently closed loop structures. Moreover, circRNAs are tissue- and developmental-specific and are dynamically expressed in different pathological conditions (Werfel et al., 2016; Siede et al., 2017; Gupta et al., 2018). Due to these characteristics, they can be used as biomarkers for MI detection (Vausort et al., 2016; Zhao et al., 2017). In this review, we generalize the available knowledge on circRNAs, elucidate the roles of the circRNA-miRNA/RBP network in MI, and explore the potential value of circRNAs in MI diagnosis.

## Biogenesis and properties of circular RNAs

There are three types of circRNAs which are produced *via* different mechanisms, namely 1) exonic circRNAs (ecircRNAs or ecRNAs) (Zhang et al., 2014); 2) exon-intron circRNAs (EIciRNAs) (Li et al., 2015); and 3) circular intronic RNAs (ciRNAs) (Conn et al., 2015). EcRNAs can be generated from the lariat-driven circularization model (Jeck et al., 2013; Jeck and Sharpless, 2014), intron pairing-driven circularization model (Deininger, 2011; Jeck et al., 2013), and RBP-dependent model (Ashwal-Fluss et al., 2014; Conn et al., 2015). EIciRNAs can be produced from intron pairing-driven circularization model (Deininger, 2011; Jeck et al., 2013) and RBP-dependent mode (Ashwal-Fluss et al., 2014; Conn et al., 2015). CiRNAs can be formed *via* a single pathway, which requires the binding of introns and back-splicing of the spliceosome (Zhang et al., 2013). The majority of circRNAs are ecRNAs (Hansen et al., 2013; Memczak et al., 2013). Notably, ecRNAs are dominantly located in the cytoplasm (Zhang et al., 2020c). Due to intron sequences, EIciRNAs and ciRNAs are confined to the nucleus, implying their different functional mechanisms (Zhang et al., 2020c).

CircRNAs have some common biological properties, such as high stability (Suzuki et al., 2006), wide distribution (Jeck et al., 2013; Zheng et al., 2016; Xu et al., 2017; Zeng et al., 2017), and expression specificity (Jakobi et al., 2016; Li et al., 2017; Xu et al., 2017). These features indicate the multifunctional roles of circRNAs in various biological processes.

## Functional mechanisms of circular RNAs

**FIGURE 1:**
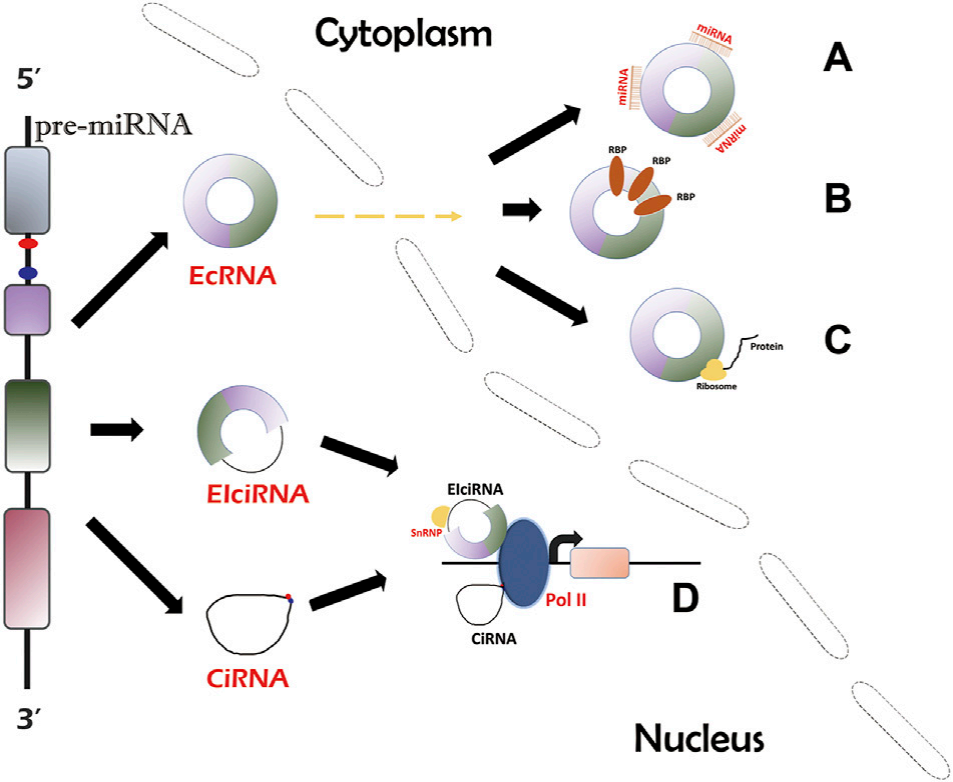
Biogenesis and circRNA action mechanisms. There are three types of circRNAs. EcRNAs can be transported into cytosol and account for the majority of circRNAs, while EIciRNAs and ciRNAs are confined to the nucleus. EcRNAs can act as miRNA/RBP sponges **(A,B)** and protein translation templates **(C)**. EIciRNAs and ciRNAs can function by regulating parental gene transcription **(D)**.

Many studies have illustrated the general mechanisms of actions of circRNAs (Zhang et al., 2013; Li et al., 2015; Legnini et al., 2017; Yang et al., 2018; Zhang et al., 2020c). EcRNAs contain miRNA response elements (MREs) that help ecRNAs absorb miRNAs like sponges. One ecRNA can bind to differernt number of miRNAs depending on the MRE number. The sponging effect inhibits the activity of functional miRNAs and thus upregulates miRNA targets (Hansen et al., 2013; Garikipati et al., 2019) (Figure 1A). In addition, ecRNAs can function by sponging RBPs and suppressing RBP activity, thereby acting on downstream pathways (Zhang et al., 2021b) (Figure 1B). Moreover, some ecRNAs have been revealed to encode peptides (Legnini et al., 2017; Yang et al., 2018) (Figure 1C). CircRNAs lack typical translation initiation structures (5′ cap and 3′ polyadenylated tail). However, some special elements, such as the internal ribosome entry site and N-methyladenosine, can be used to facilitate translational initiation of ecRNAs (Wesselhoeft et al., 2018). CiRNAs and EIciRNAs can serve as transcriptional regulators of the parental genes (Zhang et al., 2013; Li et al., 2015) (Figure 1D). CiRNAs directly bind to RNA polymerase II (Pol II) to promote transcription (Zhang et al., 2013). EIciRNAs indirectly interact with Pol II through binding to the U1 small nuclear ribonucleoproteins (snRNPs) (Li et al., 2015). CircRNAs participate in MI mainly by acting as sponges of miRNAs/RBPs (Garikipati et al., 2019; Si et al., 2020; Wang Y. et al., 2021; Ye et al., 2021; Zhu et al., 2021) (Table 1).

**TABLE 1:** CircRNAs with MI.

CircRNAs	Source	Target	Action mechamism	Regulation	References
*circRNAs that aggravate the syptoms caused by MI*					
circNfix	Mouse heart, CM	miR-214	miR-214-Gsk3β	Suppressing cardiac regeneration and functional recovery	Huang et al. (2019)
circPostn	MI patients, CM mouse heart	miR-96-5p	miR-96-5p-BNIP3	Aggravating myocardial injury and cardiac remodeling	Cheng et al. (2020)
circPAN3	Rat heart	miR-221	miR-221-FoxO3-ATG7	Promoting cardiac fibrosis induced by MI	Li et al. (2020)
circ_0060745	Mouse heart	---	NF-κB	Promoting CM apoptosis and inflammation, increasing infarct size	Zhai et al. (2020)
circFASTKD1	HCMEC	miR-106a	miR-106a-LATS1/2-YAP	Suppressing cardiac function and cardiac repairment	Gao et al. (2021)
circHIPK3	Mouse heart, CM	miR-93-5p	miR-93-5p-Rac1-PI3K/Akt<	Enhancing cardiac dysfunction	Wu et al. (2021)
circRNA 010567	CM	miR-141	miR-141-DAPK1	Deteriorating the CM injury	Zhao et al. (2021c)
	Rat heart	TGF-β1 and Smad3	TGF-β1 and Smad3	Promoting cardiac fibrosis	Bai et al. (2020)
circROBO2	Mouse heart, CM	miR-1184	miR-1184-TRADD	Promoting myocardial apoptosis	Chen et al. (2021)
MFACR	MI patients, CM, mouse heart	miR-125b	miR-125b	Promoting the apoptosis of CMs	Wang et al. (2021b)
circ-NNT	MI patients, CM	miR-33a-5p	miR-33a-5p-USP46	Promoting pyroptosis and aggravate myocardial I/R injury	Ye et al. (2021)
*circRNAs that attenuate the symptoms caused by MI*					
circFndc3b	Mouse heart, MCEC, HUVEC	FUS	FUS- VEGFA	Attenuating apoptosis, improving the functions of left ventricular, and promoting neovascularization	Garikipati et al. (2019)
circ-Ttc3	Rat heart, CM	miR-15b	miR-15b-Arl2	Inhibiting CM apoptosis and cardiac dysfunction	Cai et al. (2019)
circCDYL	Mouse heart, CM	miR-4793-5p	miR-4793-5p-APP	Promoting CM proliferation, cardiac regeneration and repairment	Zhang et al. (2020d)
circHIPK3	Mouse heart, CM, HCAEC	miR-133a	miR-133a- CTGF	Promoting myocardial regeneration and improving myocardial function	Zhang et al. (2020d)
	CMs, cardiac endothelial cell	miR-29	miR-29-VEGFA	Improving cardiac function and suppressing cardiac fibrosis	Wang et al. (2020)
circMACF1	Mouse heart, CM	miR-500b-5p	miR-500b-5p -EMP1	Attenuating AMI symptoms	Zhao et al. (2021a)
circSNRK	Rat heart, CM	miR-103-3p	miR-103-3p-SNRK-GSK3β	Promoting angiogenesis and improving cardiac functions	Zhu et al. (2021)
circ_0001206	CM	miR-665	miR-665-CRKL	Alleviating H/R-induced CM injury	Wang et al. (2022)
circFoxo3	Rat heart, CM	KAT7	KAT7 -HMGB1	Ameliorating cardiac dysfunction and attenuating autophagy	Sun et al. (2021)

## CircRNAs and MI

### CircRNAs deteriorate myocardial infarction symptoms

#### Cdr1as

Cdr1as (or CiRS-7) is one of the most studied circRNAs that functions as a miR-7a/b sponge or inhibitor (Hansen et al., 2011; Hansen et al., 2013; Memczak et al., 2013). Geng et al. investigated the role of the Cdr1as-miR-7a pathway in MI-induced apoptosis Geng et al. (2016). The levels of Cdr1as and miR-7a increased in MI models *in vivo* and *in vitro*. Cdr1as upregulation enhanced cell apoptosis, while miR-7a overexpression reversed the effect. SP1 and PARP were identified as new miR-7a targets. SP1 is a transcription factor involved in hypoxic gene transcription (Eltzschig et al., 2009), while PARP functions in I/R-induced apoptosis (Li et al., 2014). SP1 and PARP overexpression inhibited the cell apoptosis induced by miR-7a. Overexpression of Cdr1as enlarged the cardiac infarct size and increased SP1 and PARP levels, while miR-7a overexpression reversed these trends (Geng et al., 2016). In general, Cdr1as can promote MI *via* the miR-7a-SP1/PARP axis (Geng et al., 2016).

#### CircNfix

Superenhancers are active enhancers that are enriched for binding key master transcription factors (Whyte et al., 2013). Nfix circRNA (circNfix) was validated to be a superenhancer-associated circRNA (SE-circRNA) (Huang et al., 2019). CircNfix was highly expressed in adult humans, rats, and mice hearts (Huang et al., 2019). CircNfix overexpression significantly inhibited cardiomyocyte (CM) proliferation and enlarged the infarct size, blocking cardiac regeneration. On the other hand, downregulation of circNfix promoted CM proliferation and angiogenesis, inhibited post-MI apoptosis, and relieved cardiac dysfunction (Huang et al., 2019). Analyses showed that circNfix might interact with Ybx1 whose level could be regulated by Nedd4l (an E3 ubiquitin ligase). CircNfix promoted the interaction between Ybx1 and Nedd4l, inducing Ybx1 degradation by ubiquitination.

These results suggested that circNfix might suppress CM proliferation by reinforcing ubiquitin-dependent degradation of Ybx1 (Huang et al., 2019). CircNfix was found to have three binding sites with miR-214. Dual-luciferase assays, RNA fluorescence *in situ* hybridization assays, and RNA pull-down assays validated the binding of miR-214 with circNfix (Huang et al., 2019). MiR-214 was then found to interact with the 3′UTR of glycogen synthase kinase 3β (Gsk3β) in a highly conserved site among species. MiR-214 overexpression decreased the Gsk3β level. Gsk3β regulates CM proliferation by degrading β-catenin (D'Uva et al., 2015). Downregulation of circNfix increased miR-214 levels, thereby reducing Gsk3β expression (Huang et al., 2019). A miR-214 inhibitor diminished the suppressive effect of circNfix knockdown on Gsk3β. No apparent relationship was observed between Ybx1 and miR-214. Thus, circNfix might suppress cardiac regeneration and functional recovery after MI through two independent pathways: enhancement of ubiquitin-dependent Ybx1 degradation and inhibition of miR-124 activity (Huang et al., 2019).

#### CircPostn

CircPostn was upregulated in MI patient plasma and MI models (hypoxia and reoxygenation (H/R)-treated cell models and mouse models) (Cheng et al., 2020). The knockout of circPostn attenuated myocardial injury and cardiac remodeling caused by MI in mice (Cheng et al., 2020). CircPostn could alter the expression of myocardial fibrosis and remodeling markers. Bioinformatic analysis and the dual-luciferase reporter assay revealed the interaction between circPostn and miR-96-5p. MiR-96-5p levels were decreased in H/R-treated CMs. The analysis also validated that miR-96-5p could interact with BNIP3 (Cheng et al., 2020). BNIP3 belongs to the Bcl-2 family and mediates non-apoptotic/apoptotic cell death (Chinnadurai et al., 2008; Burton and Gibson, 2009). BNIP3 plays a critical role in HF, especially during ischemia (Webster et al., 2005). CircPostn knockdown decreased BNIP3 expression, whereas miR-96-5p knockdown attenuated this effect, indicating that circPostn can promote BNIP3 expression *via* downregulating miR-96-5p (Cheng et al., 2020). In general, circPostn can aggravate MI-induced myocardial injury and cardiac remodeling by the miR-96-5p-BNIP3 axis (Cheng et al., 2020).

#### CircPAN3

CircPAN3 is produced from the *PAN3* gene, which is involved in the stem cell renewal (Zhu et al., 2019) and drug resistance (Shang et al., 2019). Li et al. investigated the role of circPAN3 in MI-induced myocardial fibrosis Li et al. (2020). In the MI rat model, the fibrotic markers were significantly upregulated, implying the occurrence of myocardial fibrosis. Notably, circPAN3 levels were increased in MI heart tissues (Li et al., 2020). CircPAN3 knockdown alleviated myocardial fibrosis *in vivo* and *in vitro*. Bioinformatic analyses and the dual-luciferase reporter assay validated the interaction between circPAN3 and miR-221. MiR-221 expression was markedly decreased in MI tissues. Overexpression of miR-221 inhibited autophagy and myocardial fibrosis. CircPAN3 overexpression decreased miR-221 level, whereas circPAN3 downregulation increased the level (Li et al., 2020). MiR-221 was found to interact with FoxO3 and negatively regulated its function (Li et al., 2020). FoxO3 was testified to directly bind to ATG7 and promote its activity. FoxO3 and ATG7 were highly expressed in MI heart tissues. MiR-221 mimic suppressed FoxO3 and ATG7 expression. In general, circPAN3 participates in MI-induced myocardial fibrosis by the miR-221-FoxO3-ATG7 axis (Li et al., 2020).

#### Circ_0060745

Zhai et al. reported the role of circ_0060745 in acute MI (AMI) Zhai et al. (2020). Circ_0060745 expression was increased in the infarcted myocardium of AMI mice. Particularly, circ_0060745 level was enriched in myocardial fibroblasts. Knockdown of circ_0060745 reduced the infarct size of the AMI mouse heart, whereas circ_0060745 overexpression increased it (Zhai et al., 2020). Circ_0060745 downregulation partially improved the impaired cardiac function of AMI mice. These results suggested the regulatory role of circ_0060745 in cardiac function. Circ_0060745 knockdown significantly attenuated CM apoptosis in the infarcted areas and inhibited CM apoptosis under hypoxia. In cardiac fibroblasts under hypoxia, knockdown of circ_0060745 lowered the migratory capability of primary peritoneal macrophages and reduced the expression levels of proinflammatory cytokines (IL-6, IL-12, IL-1β and TNF-α) (Zhai et al., 2020). Proinflammatory cytokine levels can be increased by activating NF-κB (Liu et al., 2017). Subsequent analysis showed that circ_0060745 knockdown in cardiac fibroblasts under hypoxia suppressed NF-κB activation. These data suggest that circ_0060745 may participate in AMI by regulating NF-κB activation. However, the exact mechanisms remain unclear and should be explored in further studies (Zhai et al., 2020).

#### CircFASTKD1

CircFASTKD1 is involved in the pathogenesis of angiogenesis after MI (Gao et al., 2021). Gao et al. found that downregulation of circFASTKD1 enhanced HCMEC viability Gao et al. (2021). CircFASTKD1 overexpression in HUVECs exhibited significantly reduced cell viability and led to relatively weaker angiogenic ability. In addition, circFASTKD1 overexpression suppressed the migration and mobility of vascular endothelial cells. These data suggested that circFASTKD1 might repress angiogenesis in vascular endothelial cells. Further analyses revealed that circFASTKD1 could bind to miR-106a and directly suppress miR-106a expression. Overexpression of miR-106a could reverse the effects of circFASTKD1 on angiogenesis. Large tumor suppressor kinase 1 (LATS1) and LATS2, the upstream kinases of Yes-associated protein (YAP) in the classical hippo/YAP pathway (Murakami et al., 2011; Li et al., 2013; Yao et al., 2015), were revealed to be the direct targets of miR-106a. CircFASTKD1 overexpression elevated LATS1 and LATS2 levels, hence promoting YAP phosphorylation. The aforementioned results showed that circFASTKD1 inhibited angiogenesis through the miR-106a/LATS1/2/YAP pathway (Gao et al., 2021). The function of circFASTKD1 under hypoxic conditions was also validated. Under hypoxic conditions, circFASTKD1 overexpression reduced the mobility, viability, migration and tube formation of vascular endothelial cells, whereas circFASTKD1 downregulation promoted angiogenesis. *In vivo* experiments showed that circFASTKD1 knockdown could improve cardiac function and promote cardiac repair after MI. These findings demonstrate the inhibitory role of circFASTKD1 in the angiogenesis after MI by the miR-106a-LATS1/2/YAP axis (Gao et al., 2021).

#### CircHIPK3

CircHIPK3 is generated from the second intron of the *HIPK3* gene (Zhang et al., 2021a). Wu et al. investigated circHIPK3 function in MI-induced cardiac dysfunction Wu et al. (2021). CircHIPK3 levels were increased in animals and the MI cell model. CircHIPK3 knockdown suppressed CM apoptosis after MI and improved cardiac function (Wu et al., 2021). Further analyses revealed that circHIPK3 could act as ceRNA to absorb miR-93-5p. MiR-93-5p could interact with RAS-related C3 botulinum toxin substrate 1 (Rac1) protein, which has been found to promote CM injury during myocardial I/R (Henninger et al., 2019). PI3K/AKT are the downstream effectors of Rac1 (Qu et al., 2018; Henninger et al., 2019; Zhang et al., 2020b), and PI3K/AKT pathway activation is related to myocardial injury (Hauselmann et al., 2011; Zhang and Cui, 2018). MiR-93-5p overexpression reversed the detrimental effect of circHIPK3 on MI-induced CM injury. In general, circHIPK3 could suppress miR-93-5p levels and activate the Rac1-PI3K/Akt pathway, thereby enhancing MI-induced cardiac dysfunction (Wu et al., 2021).

#### CircRNA 010567

Zhao et al. explored the role of circRNA 010567 in hypoxia-induced MI Zhao Q. et al. (2021). An *in vitro* MI model was established with H9c2 cells under hypoxic conditions. CircRNA 010567 levels were increased in hypoxia-induced H9c2 cells (Zhao Q. et al., 2021). The analytical results showed that circRNA 010567 could bind to miR-141. MiR-141 expression was downregulated in hypoxia-induced H9c2 cells. CircRNA 010567 silencing increased miR-141 levels. Furthermore, circRNA 010567 knockdown and miR-141 overexpression increased H9c2 cell viability, reduced apoptosis, and suppressed caspase-3 activity (Zhao Q. et al., 2021). MiR-141 was found to interact with death-associated protein kinase-1 (DAPK1) which is related to I/R injury (Yu et al., 2021). DAPK1 was upregulated in hypoxia-induced H9c2 cells. DAPK1 overexpression reversed the miR-141 effect on cell viability and apoptosis. Therefore, circRNA 010567 may deteriorate CM injury by the miR-141-DAPK1 axis in the *in vitro* MI model (Zhao Q. et al., 2021).

In rats, Bai et al. investigated the role of circRNA 010567 in MI-induced myocardial fibrosis Bai et al. (2020). In MI rats, circRNA 010567 knockdown significantly improved cardiac function. In addition, circRNA 010567 downregulation decreased myocardial apoptosis, facilitated the orderly arrangement of myocardial cells, and alleviated myocardial interstitial fibrosis. TGF-β1 plays a critical role in fibrosis processes and is positively correlated with the pathogenesis of myocardial fibrosis (Sun et al., 2015). The expression levels of TGF-β1 and Smad3 were elevated in MI rat heart tissuess, which were substantially suppressed by circRNA 010567 knockdown, indicating its inhibitory role in myocardial fibrosis occurrence. These results demonstrated that the normal circRNA 010567 level might promote MI-induced myocardial fibrosis (Bai et al., 2020).

#### CircROBO2

CircROBO2 levels were increased in MI models both *in vivo* and *in vitro* (CMs under hypoxia treatment) (Chen et al., 2021). CircROBO2 silencing improved cell viability and suppressed apoptosis, whereas its overexpressionhad contradictory effects (Chen et al., 2021). In MI mice, circROBO2 knockdown decreased the MI area and CK-MB and LDH levels. These results suggested that circROBO2 might promote MI pathogenesis. MiR-1184 expression was reduced in MI models. MiR-1184 acted as a circROBO2 target and could promote CM viability and repress CM apoptosis. Downregulation of circROBO2 increased miR-1184 levels and intensified its effect on CMs. Further analyses showed that miR-1184 directly interacted with the TNFR1-associated death domain protein (TRADD) (Chen et al., 2021). TRADD regulates apoptosis, participates in various signaling pathways, and plays a vital role in cardiovascular disease (CVD) (Pobezinskaya and Liu, 2012). TRADD levels were increased in MI models. MiR-1184 was found to inhibit TRADD expression. Overexpression of circROBO2 upregulated TRADD and downregulated miR-1184 levels, indicating that circROBO2 might upregulate TRADD expression by sponging miR-1184. These results demonstrat that circROBO2 promotes MI development by increasing myocardial apoptosis *via* the miR-1184-TRADD axis (Chen et al., 2021).

#### MFACR

CircRNA MFACR is involved in mitochondrial fission and cardiac apoptosis (Han et al., 2018). Wang et al. explored the role of MFACR in MI Wang S. et al. (2021). MFACR expression was significantly increased in MI patients. In MI mice, MFACR overexpression significantly aggravated myocardial injuries. MiR-125b was downregulated in MI mice. In cell models (CMs under hypoxia treatment), the same expression pattern was observed. MFACR was negatively correlated with miR-125b. MFACR was then validated to inhibit miR-125b expression by increasing the methylation of miR-125b gene. MFACR overexpression promoted the apoptosis of hypoxia-treated CMs, which could be reversed by miR-125b overexpression. In general, MFACR might promote CM apoptosis in MI by downregulating miR-125b (Wang S. et al., 2021).

#### Circ-NNT

Pyroptosis is involved in MI development in rodents (Sandanger et al., 2013). Its role in reperfusion injury in MI has been revealed in both *in vitro* (A/R CMs) and *in vivo* (I/R mice) models (Ye et al., 2021). Ye et al. studied the functions of pyroptosis in MI patients and myocardial I/R injury models Ye et al. (2021). In serum of MI patients, IL-1β and IL-18 (pyroptosis-related pro-inflammatory cytokines) showed increased concentration. The levels of IL-1β, IL-18, and pyroptosis-related inflammatory caspases (caspase-1 and 11) were elevated, indicating pyroptosis activation (Ye et al., 2021). The circRNA microarray revealed that circ-NNT was most differentially expressed. Circ-NNT was mainly expressed in the heart, suggesting its heart-specific role. Circ-NNT was upregulated in MI patients and I/R models. Circ-NNT knockdown alleviated MI symptoms in I/R mice and reduced the expression of caspase-1, caspase-11, IL-1β, and IL-18 in A/R CMs. These results indicated that circ-NNT might promote pyroptosis progression. Circ-NNT was found to sponge miR-33a-5p. MiR-33a-5p could interact with USP46. MiR-33a-5p and USP46 were also validated to be relevant to pyroptosis. Overexpression of circ-NNT increased the USP46 level through inhibiting miR-33a-5p. Therefore, circ-NNT might promote pyroptosis to exacerbate I/R injury in MI by the miR-33a-5p-USP46 axis (Ye et al., 2021).

### Circular RNAs improve the symptoms caused by Circular RNAs

#### CircFndc3b

CircFndc3b, a 215-nt circRNA generated from exons two and three of the *Fndc3b* gene, exhibited an altered expression in MI mouse hearts on the third day after MI in a circRNA microarray analysis (Garikipati et al., 2019). CircFndc3b was significantly down-regulated in the post-MI mouse hearts (Garikipati et al., 2019). The level of circFndc3b continued to decline during a 6-weeks follow-up. CircFndc3b overexpression led to increased levels of vascular endothelial growth factor A (VEGFA), a potent cardioprotective molecule (Piwecka et al., 2017) and a regulator of angiogenesis (Krishnamurthy et al., 2011). CircFndc3b upregulation reduced the H_2_O_2_-induced MCEC apoptosis and enhanced the tube formation ability of HUVECs (Garikipati et al., 2019). CircFndc3b overexpression in CMs of post-MI hearts attenuated apoptosis, improved left ventricular functions, and promoted neovascularization. Dual-luciferase reporter gene assays showed that circFndc3b might bind to miR-93-3p, miR-412-3p, and miR-298-5 (Garikipati et al., 2019). However, circFndc3b did not function through sponging these miRNAs. In silico analysis, RNA binding protein immunoprecipitation (RIP), and pull-down assays validated the interaction between circFndc3b and FUS protein. CircFndc3b overexpression suppressed the expression of FUS protein and rescued the inhibitory effect of FUS on VEGFA (Garikipati et al., 2019). In conclusion, circFndc3b plays a cardioprotective role by regulating CM apoptosis, neovascularization and infarct size. CircFndc3b might function by the FUS-VEGFA signaling pathway (Garikipati et al., 2019).

#### Circ-Ttc3

The circular RNA-tetratricopeptide repeat domain 3 (Ttc3) is a highly expressed circRNA in the mouse heart (Cai et al., 2019). Circ-Ttc3 was upregulated in the ischemic myocardium of rats with MI (Cai et al., 2019). Circ-Ttc3 silencing aggravated CM apoptosis and cardiac dysfunction after MI, suggesting that circ-Ttc3 plays a cardioprotective role (Cai et al., 2019). Circ-Ttc3 was found to bind to miR-15b-5p. Bioinformatic prediction and dual-luciferase reporter assays proved that miR-15b directly interacted with ADP-ribosylation factor-like protein 2 (Arl2) (Cai et al., 2019), consistent with a previous study (Nishi et al., 2010). MiR-15b-5p inhibited Arl2 function and Arl2 was relevant to CM viability. Overexpression of circ-Ttc3 upregulated Arl2 expression. Arl2 knockdown attenuated the protective effect of circ-Ttc3 overexpression on CM apoptosis. Thus, circ-Ttc3 might play a cardioprotective role by the miR-15b-Arl2 regulatory pathway (Cai et al., 2019).

#### CircCDYL

CircCDYL expression was decreased in MI mouse hearts and the MI cell model (Zhang M. et al., 2020). CircCDYL overexpression promoted CM proliferation and cardiac regeneration and repairment, whereas circCDYL knockdown suppressed these processes (Zhang M. et al., 2020). Bioinformatics analyses and dual-luciferase reporter assays validated the binding of circCDYL and miR-4793-5p. MiR-4793-5p has been found to be associated with delayed cerebral infarction (Lu et al., 2017). Bioinformatics tools revealed that amyloid precursor protein (APP) might be the target of miR-4793-5p (Wilkins and Swerdlow, 2017). The APP protein level was downregulated by miR-4793-5p overexpression. In brief, circCDYL might improve cardiac function after MI by the miR-4793-5p-APP pathway (Zhang M. et al., 2020).

#### CircHIPK3

Si et al. found that circHIPK3 was positively regulated by the transcription factor Gata4, which has been revealed to regulate CM proliferation and myocardial regeneration Si et al. (2020). Overexpression of circHIPK3 promoted CM and human coronary artery endothelial cell (HCAEC) proliferation (Si et al., 2020). Moreover, circHIPK3 overexpression in the MI mouse heart promoted angiogenesis, reduced the infarcted area size, and elevated cardiac pumping capacity. Knockdown of circHIPK3 led to opposite results. All data suggested that circHIPK3 might promote myocardial regeneration and improve myocardial function after MI. In HCAECs, circHIPK3 could bind to miR-133a, which is essential for cardiac development and protection (Si et al., 2020). MiR-133a could interact with the connective tissue growth factor (CTGF) which is intimately related to angiogenesis (Duan et al., 2015; Yang et al., 2017). Additional analyses validated the possible role of the circHIPK3-miR-133a-CTGF axis in the HCAEC function and angiogenesis (Si et al., 2020). However, in CMs, circHIPK3 did not act through the miRNA-mRNA pathway, but instead functioned by binding to Notch1 Intracellular Domain (N1ICD) protein (Si et al., 2020).

In another study by Wang et al., the regulatory role of exosomal circHIPK3 in cardiac angiogenesis after MI was investigated (Wang et al., 2020). Hypoxia-induced CMs could release circHIPK3-containing exosomes (HPC-exos) (Wang et al., 2020). HPC-exos could improve cardiac function and suppress cardiac fibrosis. HPC-exos rescued circHIPK3 levels under H_2_O_2_ treatment, induced tube formation, and facilitated angiogenesis. Under oxidative conditions, circHIPK3 overexpression stimulated the proliferation and migration ability of cardiac endothelial cells. CircHIPK3 was identified to sponge miR-29, which is known to regulate VEGFA (Domingues et al., 2011; Zhang et al., 2016), indicating that circHIPK3 functions through inhibiting miR-29 and upregulating VEGFA. Therefore, HPC-exos played a cardioprotective role by the miR-29-VEGFA axis (Wang et al., 2020).

#### CircMACF1

Zhao et al. explored the role of circMACF1 in AMI for the first time Zhao B. et al. (2021). CircMACF1 levels were decreased significantly in the myocardial tissue of MI mice (Zhao B. et al., 2021). CircMACF1 level was also downregulated in the hypoxia-induced cell model. Bioinformatics analyses and dual-luciferase reporter assays revealed that circMACF1 could interact with miR-500b-5p (Zhao B. et al., 2021). MiR-500b-5p was upregulated in MI models. Overexpression of circMACF1 reduced miR-500b-5p levels, whereas knockdown of circMACF1 elevated miR-500b-5p levels. Subsequent analyses found that miR-500b-5p could interact with epithelial membrane protein 1 (EMP1) that has been revealed to regulate CVD pathogenesis (Xu et al., 2011; Yu et al., 2013). Overexpression of miR-500b-5p downregulated EMP1 expression (Zhao B. et al., 2021). Thus, circMACF1 might upregulate EMP1 by sponging miR-500b-5p in MI. Overexpression of circMACF1 inhibited CM apoptosis and cardiac dysfunction caused by AMI, which could be reversed by upregulating miR-500b-5p or downregulating EMP1. Therefore, these findings indicated that circMACF1 could attenuate AMI symptoms by the miR-500b-5p-EMP1 axis (Zhao B. et al., 2021).

#### Circular RNAs SNRK

High-throughput sequencing by Zhu et al. revealed that circSNRK expression was found to be significantly reduced in the MI rat hearts Zhu et al. (2021), especially in CMs. CircSNRK upregulation ameliorated the CM function, such as reducing apoptosis and increasing CM proliferation. Moreover, circSNRK upregulation enhanced angiogenesis and improved cardiac functions, implying the cardioprotective role of circSNRK (Zhu et al., 2021). Bioinformatics analyses and dual-luciferase assays confirmed that miR-103-3p could interact with circSNRK and bind to the *SNRK* gene. MiR-103-3p is implicated in apoptosis (Leslie et al., 2018) and cell proliferation (Natarelli et al., 2018). In the myocardium of MI patients, miR-103-3p was upregulated. MiR-103-3p elevation exacerbated CM apoptosis and suppressed CM proliferation. SNRK upregulation could rescue the impaired function of circSNRK overexpression caused by miR-103-3p mimics. Overall, circSNRK exerted the cardioprotective role by the miR-103-3p-SNRK regulatory axis (Zhu et al., 2021). SNRK protein was found to regulate the phosphorylation of GSK3β, which has been shown to modulate apoptosis and proliferation (Woulfe et al., 2010). Immunoprecipitation showed the direct interaction between SNRK and GSK3β. These results demonstrated that the protective effects of SNRK in CMs might be dependent on the GSK3β pathway (Zhu et al., 2021). Hence, circSNRK played a cardioprotective role through the miR-103-3p-SNRK-GSK3β axis (Zhu et al., 2021).

#### Circ_0001206

CRK like proto-oncogene, adaptor protein (CRKL) has been found to mitigate CM injury induced by hypoxia/reoxygenation (H/R) in MI (Zhang et al., 2015). In this study, H/R treatment was used to construct an *in vitro* MI model (Wang et al., 2022). Wang et al. found a circRNA produced by the *CRKL* gene, circ_0001206 (Wang et al. (2022). Circ_0001206 was significantly downregulated in H/R-treated CMs and the MI mouse model. Circ_0001206 overexpression attenuated H/R-induced cardiac injuries in MI mice, reduced the infarct size, and promoted CM proliferation. In H/R-treated CMs, circ_0001206 overexpression enhanced cell viability and suppressed apoptosis. The results suggested the cardioprotective role of circ_0001206 (Wang et al., 2022). Circ_0001206 could bind to miR-665, which was validated to target CRKL. Circ_0001206 upregulation increased CRKL level, while miR-665 overexpression reversed this effect. Moreover, miR-665 overexpression inhibited the protective effect of circ_0001206. Taken together, circ_0001206 attenuated H/R-induced CM injury by modulating the miR-665-CRKL axis (Wang et al., 2022).

#### CircFoxo3

CircFoxo3 is derived from the *Fox O 3* gene and has been found to participate in cancers and CVD (Zhang et al., 2021b). The cardioprotective role of circFoxo3 during MI development was investigated (Sun et al., 2021). CircFoxo3 expression was reduced in MI rats. Upregulation of circFoxo3 alleviated MI-induced cardiac dysfunction and cell autophagy. Moreover, circFoxo3 overexpression repressed CM injury, apoptosis, autophagy, and inflammation induced by oxygen-glucose deprivation (OGD). CircFoxo3 inhibited KAT7 expression. KAT7 knockdown relieved OGD-induced CM injury and autophagy. In addition, knockout of KAT7 downregulated the expression of high mobility group box 1 (HMGB1), implying the positive modulation of KAT7 on HMGB1. CircFoxo3 overexpression repressed HMGB1 expression, whereas KAT7 overexpression rescued HMGB1 level. Therefore, circFoxo3 might suppress HMGB1 expression *via* inhibiting KAT7. Conclusively, circFoxo3 might play a cardioprotective role in MI by the KAT7-HMGB1 axis (Sun et al., 2021).

**FIGURE 2:**
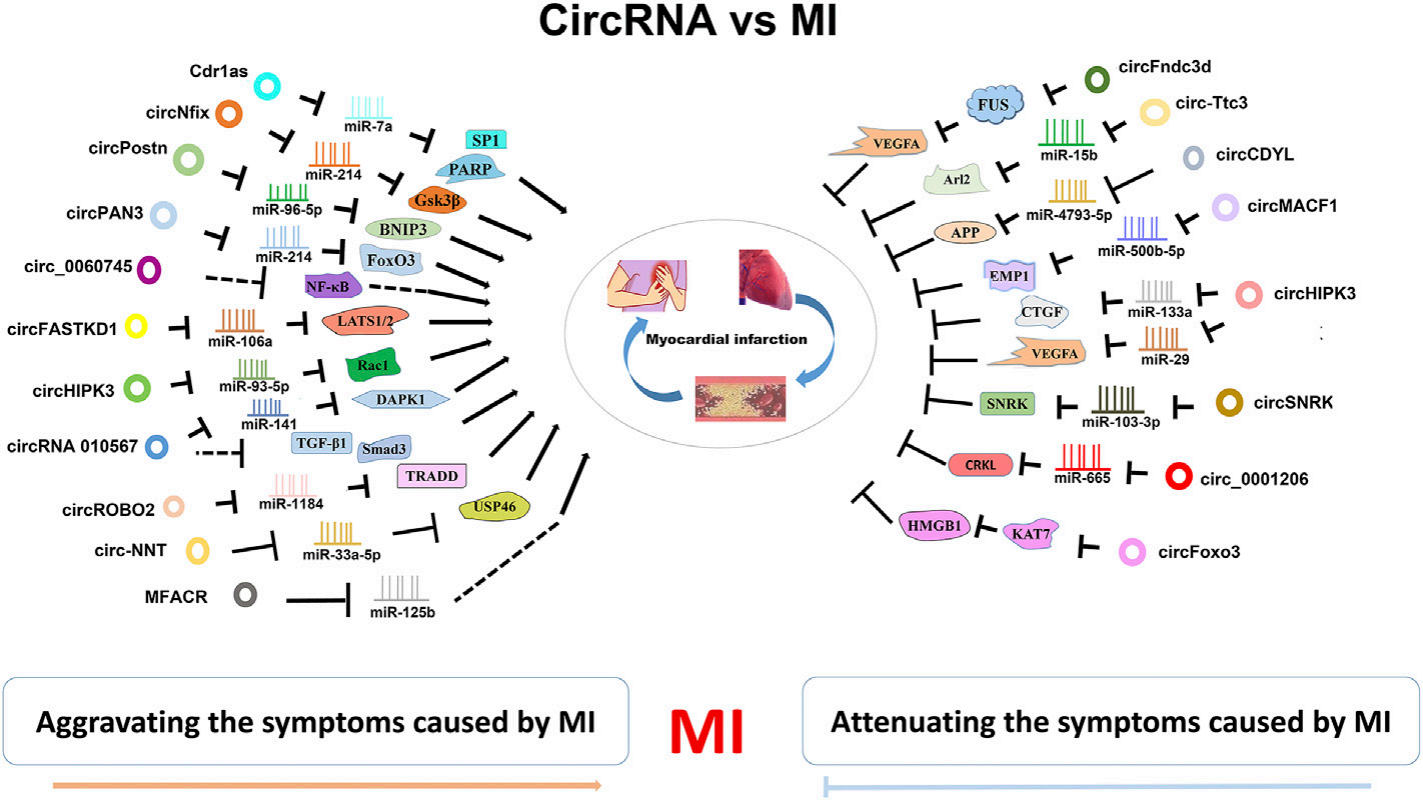
CircRNA-miRNA/RBP axies in MI. CircRNAs are widely invovled in MI. They can attenuate or aggravate the MI symptoms by inhibiting various miRNA/RBP targets.

In summary, circRNAs are important modulators for MI. Some circRNAs play cardioprotecrive role in MI models, whereas other ones play an opposite role to deteriorating MI symptoms (Figure 2; Table 1).

## Clinical significance of Circular RNAs in myocardial infarction

Currently, the most used clinical diagnostic biomarkers for MI are some proteins, such as troponin, creatine kinase-MB and myoglobin (Lobbes et al., 2010). However, they cannot be detected in the early stage of MI and are not sensitive and specific. Troponin is a biomarker of myocardial injury and can be observed in various diseases, such as severe heart failure and chronic kidney failure (Jensen et al., 2007). In addition, their levels are affected by some factors in patients, including age, genetic background, medication, and lifestyle (Chen et al., 2008), leading to poor accuracy. Noncoding RNAs have been found to have diagnostic value (Zhang et al., 2020c; Liu et al., 2021), including circRNAs. CircRNAs are highly stable, widely distributed, and tissue- and developmental stage-specific. These characteristics are valuable for biomarkers. Moreover, the levels of circRNAs in circulatory system are not low and some of them are even highly expressed, leading to easier and more accurate detection. Studies have found that circulating circRNAs might be promising biomarkers for MI (Table 2).

**TABLE 2:** Circulating circRNAs as biomarkers of MI.

CircRNA	Source	Regulation	Application	References
circRNA_081881	plasma	down	Diagnostic biomarker	Deng et al. (2016)
MICRA	whole blood	------	prognostic biomarker for MI risk stratification	Salgado-Somoza et al. (2017)
	peripheral blood	Down	Predictive biomarker of ventricular dysfunction in MI patients	Vausort et al. (2016)
circRNA_104761	whole blood	Down	Diagnostic biomarker	Yang et al. (2021)
hsa_circRNA_001654	whole blood	Up	Diagnostic biomarker	Zhao et al. (2021b)
hsa_circRNA_405624	whole blood	Up	Diagnostic biomarker	Zhao et al. (2021b)
hsa_circRNA_091761	whole blood	Up	Diagnostic biomarker	Zhao et al. (2021b)
hsa_circRNA_406698	whole blood	Up	Diagnostic biomarker	Zhao et al. (2021b)

*PPAR-gamma* has been revealed to protect heart from AMI (Chu et al., 2018). Deng et al. conducted a microarray to explore *PPAR-gamma*-related circRNAs in the plasma of AMI patients Deng et al. (2016). In 160 dynamically expressed circRNAs, circRNA_081881 was significantly downregulated in AMI patients compared with the control group. CircRNA_081881 was found to have seven binding sites for miR-548. According to bioinformatics analysis, miR-548 might interact with the *PPAR-gamma* gene. CircRNA_081881 knockdown suppressed expression of *PPAR-gamma*. The results indicated that plasma circRNA_081881 might be a biomarker for the diagnosis and therapy of AMI (Deng et al., 2016).

MI-associated Circular RNA (MICRA) has been found to have clinical importance. Salgado-Somoza et al. assessed the role of MICRA in the risk stratification after AMI by using whole blood samples (Salgado-Somoza et al., 2017). The Akaike Information Criteria (AIC) was analyzed to predict the value of MICRA in a multi-parameter clinical risk stratification model (Salgado-Somoza et al., 2017). A lower AIC indicates a better predictive value. The inclusion of MICRA into standard criteria improved the predictive ability. Through bootstrap internal validation, MICRA was identified to be an optimal predictive biomarker. Thus, MICRA might be a novel prognostic biomarker for the risk stratification of AMI patients (Salgado-Somoza et al., 2017). In another study, MICRA exhibited lower expression levels in MI patients than in healthy controls in the peripheral blood (Vausort et al., 2016). Univariate and multivariate analyses based on logistic regression were conducted. The results demonstrated that circulating MICRA has a high predictive value of left ventricular dysfunction in MI patients (Vausort et al., 2016).

Yang et al. carried out a microarray analysis (GSE169594) in four AMI patients and four mild coronary artery stenosis patients by using whole blood samples Yang et al. (2021). Three circRNAs (circRNA_068655, circRNA_104761, and circRNA_104765) were validated to be significantly downregulated (Yang et al., 2021). Notably, circRNA_104761 was revealed to sponge microRNA-449 and microRNA-34a that is relevant to AMI (Fan et al., 2013; Zhang M. et al., 2019). In a larger cohort, circRNA_104761 levels were the lowest in AMI patients and highest in healthy coronary artery volunteers, suggesting the abundant expression of circRNA_104761 in human blood (Yang et al., 2021). ROC curve analyses showed that circRNA_104761 had high sensitivity and specificity. These findings suggested the possible diagnostic role of circRNA_10476 in AMI (Yang et al., 2021).

Zhao et al. performed the circRNA microarray with the whole blood of AMI patients Zhao et al. (2021b). More than 100 circRNAs were found to be expressed dynamically. The levels of hsa_circRNA_001654, hsa_circRNA_405624, hsa_circRNA_091761, and hsa_circRNA_406698 were significantly upregulated in the blood of AMI patients (Zhao et al., 2021b). These four circRNAs were then identified to sponge miRNAs to regulate AMI pathogenesis, indicating their potential roles as biomarkers in AMI diagnosis (Zhao et al., 2021b).

Taken together, these studies have highlighted that circulating circRNAs might act as biomarkers for MI diagnosis and prognosis.

## Conclusion and future perspective

All findings confirmed that circRNAs play essential roles in MI mainly by sponging miRNAs. CircRNAs exist in almost all eukaryotic organisms including plants and animals. They are considerably more stable than linear noncoding RNAs in circulatory system. Due to their advantages, circRNAs have great potential to be biomarkers for MI.

In clinical practice, coronary angiography is the golden standard for MI diagnosis. But it is invasive and conditional. Compared with coronary angiography, biochemical markers are non-invasive and innocuous. However, the current detection method for biochemical markers is not fast enough and can only be used in hospital or some labs. There have been some household test strips, such as early pregnancy and blood sugar rapid test strips. Only a few drops of urine or blood are needed in the test. These test strip methods cost only several seconds and the results are easy to estimate. People can conduct an initial diagnosis by themselves. The test strip method has been applied in COVID-19 diagnosis. Thus, it is expected that diagnostic strips based on circRNAs can also be prepared to improve clinical diagnosis efficiency. Moreover, it is faster and more convenient to use biological probes (gold nanocomposite probe and bioluminescence probe) compared with RT-PCR. Therefore, it may be one effective clinical diagnostic practice to construct composite probes based on circRNAs and produce rapid test kits or test strips. In addition to being disease diagnostic biomarkers, circRNAs have some other functions. CircMAPK14 can encode a peptide that might be used to prepare targeted anticancer drugs (Wang L. et al., 2021). Some patents have revealed that circRNAs can be vectors for RNA vaccine preparation. In general, circRNAs have high clinical value.

However, the utility of circRNAs as clinical biomarkers or targets may be premature due to some existing problems. First, the insufficient samples in many studies might result in deviation of experimental results, thus leading to inaccurate conclusions. Second, there are no unified experimental methods for measuring circulating circRNAs, which makes the conclusions drawn in different studies inconsistent. Third, the mechanisms of action of circRNAs are still inadequate. CircRNAs can both mediate and exacerbate MI injury. More efforts should be taken to unveil whether they are markers or mediators.

In conclusion, circRNAs are closely related to MI and might be promising diagnostic and prognostic biomarkers for MI. These findings provide new possibilities for the prevention and therapeutic intervention of MI in the future.
